# Cowper's Syringocele Causing Painful Haematuria in an Adult

**DOI:** 10.1100/tsw.2004.82

**Published:** 2004-06-07

**Authors:** Matthew B.K. Shaw, Owen Cole, Timothy R. Terry

**Affiliations:** Leicester General Hospital, Leicester, UK

## Abstract

A Cowper's syringocele in an adult is rare. Ten cases are reported in the world literature. The authors report a case of painful haematuria due to the presence of a Cowper's syringocele in an adult. The classification of lesions of the Cowper's gland is discussed together with common symptoms and differential diagnosis.

## CASE REPORT

A 39-year-old man presented with a 12-month history of post-micturition dribbling and 2 months of painful haematuria. Clinical examination revealed fullness in the perineum overlying the bulb of the urethra. Digital rectal examination demonstrated a smooth prostate gland of approximately 30 grams. Microbiological analysis of a mid-stream urine specimen was normal. His free peak flow voiding rate was 30 millilitres per second on uroflowmetry. Flexible cystourethroscopy revealed an opening in the ventral aspect of the proximal membranous urethra. This was confirmed by an ascending urethrogram and a micturating cystogram [see figures 1-2]. These demonstrate the opening to be the distal portion of a syringocele of the Cowper's gland. The patient was taught post-micturition urethral compression and his symptoms of post-micturition dribbling and haematuria resolved.

## DISCUSSION

Cowper's gland is an accessory sexual organ that contributes to semen coagulation and urethral lubrication.(1) The two main Cowper's glands are situated within the urogenital diaphragm, with a second pair of accessory glands situated in the bulbous spongiosal tissue. The main Cowper's ducts enter the ventral surface of the bulbar urethra near the midline by piercing the spongiosum. The accessory ducts can enter the urethra directly or drain into the main duct.

Lesions of the Cowper's gland have been classified by Maizels et al (2) into four groups: 1) simple syringocele a minimally dilated duct, 2) perforate syringocele -a bulbous duct that drains into the urethra and appears as a diverticulum, 3) imperforate syringocele a bulbous duct that resembles a submucosal

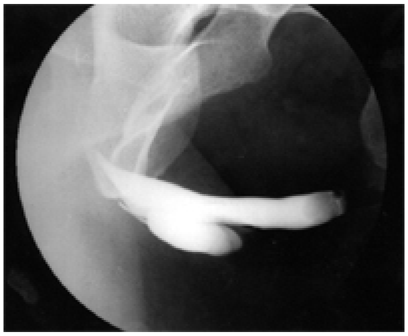

**FIGURE 1**. Ascending urethrogram showing filling of Cowper's duct.

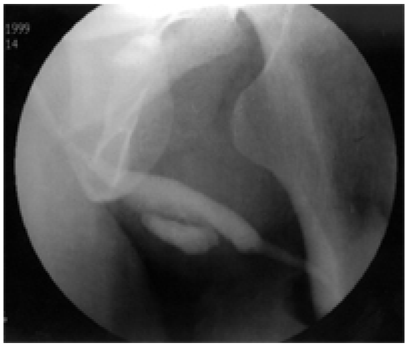

**FIGURE 2.** Micturating cystourethrogram showing a syringocele of Cowper's gland.

cyst and appears as a radiolucent mass, and 4) ruptured syringocele the membrane remaining in the urethra after a dilated duct ruptures.

Most Cowper's gland duct lesions are congenital and present in children,(3,4) with reports of cystograms in boys showing an incidence of 1.5%. Most are asymptomatic but some cause irritative and obstructive symptoms.(4) Lesions are rare in adults, (4-6) with just ten previously reported cases. The differential diagnosis of a Cowper's syringocele includes urethral duplication and urethral diverticulum. Treatment for symptomatic Cowper's syringocele not responding to conservative measures is by endoscopic deroofing.

Young adults with persistent post-micturition dribbling and other urethral symptoms should be suspected of having a Cowper's syringocele and investigated with this in mind.
